# Supplementary material for: Unveiling the Bioactive Potential of the Invasive Jellyfish Phyllorhiza punctata Through Integrative Transcriptomic and Proteomic Analyses
Source: Biomolecules. 2025 Aug 4;15(8):1121. doi: 10.3390/biom15081121 (PMC12383608; doi:10.3390/biom15081121)
Supplement: Supplementary file 1 [file biomolecules-15-01121-s001.zip › Suplementary Material/FileS3/PEPFOLD4/XP_065058056_5537/clusters.html]

 PEP-FOLD clusters


Download archive of all models  here   

|  |  |  |
| --- | --- | --- |
| # | file | sOPEP |
| # cluster 1 - Size: 2 |  |  |
|  | XP\_065058056\_5537-model1 | -40.7827 |
|  | XP\_065058056\_5537-model1.1 | -33.2357 |
| # cluster 2 - Size: 8 |  |  |
|  | XP\_065058056\_5537-model2 | -40.15 |
|  | XP\_065058056\_5537-model2.1 | -39.006 |
|  | XP\_065058056\_5537-model2.2 | -37.909 |
|  | XP\_065058056\_5537-model2.3 | -37.3522 |
|  | XP\_065058056\_5537-model2.4 | -37.1389 |
|  | XP\_065058056\_5537-model2.5 | -34.9831 |
|  | XP\_065058056\_5537-model2.6 | -31.1083 |
|  | XP\_065058056\_5537-model2.7 | -30.7513 |
| # cluster 3 - Size: 4 |  |  |
|  | XP\_065058056\_5537-model3 | -38.0507 |
|  | XP\_065058056\_5537-model3.1 | -34.7856 |
|  | XP\_065058056\_5537-model3.2 | -23.4994 |
|  | XP\_065058056\_5537-model3.3 | -17.9578 |
| # cluster 4 - Size: 2 |  |  |
|  | XP\_065058056\_5537-model4 | -36.5281 |
|  | XP\_065058056\_5537-model4.1 | -31.0014 |
| # cluster 5 - Size: 5 |  |  |
|  | XP\_065058056\_5537-model5 | -35.673 |
|  | XP\_065058056\_5537-model5.1 | -29.5858 |
|  | XP\_065058056\_5537-model5.2 | -27.6593 |
|  | XP\_065058056\_5537-model5.3 | -27.3368 |
|  | XP\_065058056\_5537-model5.4 | -21.9338 |
| # cluster 6 - Size: 2 |  |  |
|  | XP\_065058056\_5537-model6 | -35.5985 |
|  | XP\_065058056\_5537-model6.1 | -35.2174 |
| # cluster 7 - Size: 1 |  |  |
|  | XP\_065058056\_5537-model7 | -34.5646 |
| # cluster 8 - Size: 2 |  |  |
|  | XP\_065058056\_5537-model8 | -33.8873 |
|  | XP\_065058056\_5537-model8.1 | -21.2154 |
| # cluster 9 - Size: 1 |  |  |
|  | XP\_065058056\_5537-model9 | -33.0489 |
| # cluster 10 - Size: 4 |  |  |
|  | XP\_065058056\_5537-model10 | -32.7713 |
|  | XP\_065058056\_5537-model10.1 | -28.417 |
|  | XP\_065058056\_5537-model10.2 | -25.027 |
|  | XP\_065058056\_5537-model10.3 | -21.8109 |
| # cluster 11 - Size: 1 |  |  |
|  | XP\_065058056\_5537-model11 | -32.2853 |
| # cluster 12 - Size: 2 |  |  |
|  | XP\_065058056\_5537-model12 | -31.7395 |
|  | XP\_065058056\_5537-model12.1 | -16.3322 |
| # cluster 13 - Size: 2 |  |  |
|  | XP\_065058056\_5537-model13 | -30.0933 |
|  | XP\_065058056\_5537-model13.1 | -27.6907 |
| # cluster 14 - Size: 1 |  |  |
|  | XP\_065058056\_5537-model14 | -29.6076 |
| # cluster 15 - Size: 2 |  |  |
|  | XP\_065058056\_5537-model15 | -29.4996 |
|  | XP\_065058056\_5537-model15.1 | -29.2462 |
| # cluster 16 - Size: 1 |  |  |
|  | XP\_065058056\_5537-model16 | -29.4006 |
| # cluster 17 - Size: 3 |  |  |
|  | XP\_065058056\_5537-model17 | -29.3489 |
|  | XP\_065058056\_5537-model17.1 | -24.6953 |
|  | XP\_065058056\_5537-model17.2 | -15.8257 |
| # cluster 18 - Size: 1 |  |  |
|  | XP\_065058056\_5537-model18 | -28.6784 |
| # cluster 19 - Size: 1 |  |  |
|  | XP\_065058056\_5537-model19 | -28.6781 |
| # cluster 20 - Size: 1 |  |  |
|  | XP\_065058056\_5537-model20 | -28.63 |
| # cluster 21 - Size: 4 |  |  |
|  | XP\_065058056\_5537-model21 | -28.5297 |
|  | XP\_065058056\_5537-model21.1 | -28.4344 |
|  | XP\_065058056\_5537-model21.2 | -28.3268 |
|  | XP\_065058056\_5537-model21.3 | -26.6449 |
| # cluster 22 - Size: 1 |  |  |
|  | XP\_065058056\_5537-model22 | -27.7362 |
| # cluster 23 - Size: 3 |  |  |
|  | XP\_065058056\_5537-model23 | -27.6718 |
|  | XP\_065058056\_5537-model23.1 | -19.0429 |
|  | XP\_065058056\_5537-model23.2 | -14.7226 |
| # cluster 24 - Size: 7 |  |  |
|  | XP\_065058056\_5537-model24 | -27.1333 |
|  | XP\_065058056\_5537-model24.1 | -26.1483 |
|  | XP\_065058056\_5537-model24.2 | -25.8884 |
|  | XP\_065058056\_5537-model24.3 | -25.5932 |
|  | XP\_065058056\_5537-model24.4 | -25.388 |
|  | XP\_065058056\_5537-model24.5 | -23.853 |
|  | XP\_065058056\_5537-model24.6 | -22.7569 |
| # cluster 25 - Size: 1 |  |  |
|  | XP\_065058056\_5537-model25 | -27.0175 |
| # cluster 26 - Size: 2 |  |  |
|  | XP\_065058056\_5537-model26 | -26.7019 |
|  | XP\_065058056\_5537-model26.1 | -26.5392 |
| # cluster 27 - Size: 1 |  |  |
|  | XP\_065058056\_5537-model27 | -26.6856 |
| # cluster 28 - Size: 2 |  |  |
|  | XP\_065058056\_5537-model28 | -26.2893 |
|  | XP\_065058056\_5537-model28.1 | -13.0256 |
| # cluster 29 - Size: 3 |  |  |
|  | XP\_065058056\_5537-model29 | -26.0069 |
|  | XP\_065058056\_5537-model29.1 | -23.5223 |
|  | XP\_065058056\_5537-model29.2 | -20.3396 |
| # cluster 30 - Size: 1 |  |  |
|  | XP\_065058056\_5537-model30 | -25.5777 |
| # cluster 31 - Size: 1 |  |  |
|  | XP\_065058056\_5537-model31 | -25.447 |
| # cluster 32 - Size: 5 |  |  |
|  | XP\_065058056\_5537-model32 | -24.165 |
|  | XP\_065058056\_5537-model32.1 | -23.5082 |
|  | XP\_065058056\_5537-model32.2 | -21.3953 |
|  | XP\_065058056\_5537-model32.3 | -21.2904 |
|  | XP\_065058056\_5537-model32.4 | -16.5106 |
| # cluster 33 - Size: 1 |  |  |
|  | XP\_065058056\_5537-model33 | -24.1223 |
| # cluster 34 - Size: 1 |  |  |
|  | XP\_065058056\_5537-model34 | -23.1203 |
| # cluster 35 - Size: 1 |  |  |
|  | XP\_065058056\_5537-model35 | -22.8006 |
| # cluster 36 - Size: 1 |  |  |
|  | XP\_065058056\_5537-model36 | -22.2797 |
| # cluster 37 - Size: 1 |  |  |
|  | XP\_065058056\_5537-model37 | -21.4464 |
| # cluster 38 - Size: 1 |  |  |
|  | XP\_065058056\_5537-model38 | -20.8816 |
| # cluster 39 - Size: 1 |  |  |
|  | XP\_065058056\_5537-model39 | -20.3124 |
| # cluster 40 - Size: 1 |  |  |
|  | XP\_065058056\_5537-model40 | -14.8997 |
| # cluster 41 - Size: 1 |  |  |
|  | XP\_065058056\_5537-model41 | -14.6796 |
| # cluster 42 - Size: 1 |  |  |
|  | XP\_065058056\_5537-model42 | -13.7021 |
| # cluster 43 - Size: 1 |  |  |
|  | XP\_065058056\_5537-model43 | -13.4283 |
| # cluster 44 - Size: 2 |  |  |
|  | XP\_065058056\_5537-model44 | -13.1423 |
|  | XP\_065058056\_5537-model44.1 | -13.0042 |
| # cluster 45 - Size: 1 |  |  |
|  | XP\_065058056\_5537-model45 | -13.0435 |
| # cluster 46 - Size: 1 |  |  |
|  | XP\_065058056\_5537-model46 | -13.017 |
| # cluster 47 - Size: 1 |  |  |
|  | XP\_065058056\_5537-model47 | -12.8773 |
| # cluster 48 - Size: 1 |  |  |
|  | XP\_065058056\_5537-model48 | -12.8455 |
| # cluster 49 - Size: 1 |  |  |
|  | XP\_065058056\_5537-model49 | -12.5339 |
| # cluster 50 - Size: 1 |  |  |
|  | XP\_065058056\_5537-model50 | -12.3713 |
| # cluster 51 - Size: 2 |  |  |
|  | XP\_065058056\_5537-model51 | -11.5371 |
|  | XP\_065058056\_5537-model51.1 | -11.2854 |
| # cluster 52 - Size: 1 |  |  |
|  | XP\_065058056\_5537-model52 | -11.3156 |
| # cluster 53 - Size: 1 |  |  |
|  | XP\_065058056\_5537-model53 | -11.2324 |
|  |  |  |
